# Finding clues for Alzheimer's disease in online language

**DOI:** 10.1002/alz70856_105272

**Published:** 2026-01-07

**Authors:** Shira Darchi, Yuval Pinter, Talya Sadeh

**Affiliations:** ^1^ Ben Gurion University of the Negev, Beer Sheva, Israel, Israel; ^2^ Ben Gurion University of the Negev, Beer Sheva, 8410501, Israel

## Abstract

**Background:**

Natural Language Processing (NLP) has shown promise in identifying linguistic markers of MCI and Alzheimer's Disease (AD). Prior studies have achieved classification accuracy of 80%‐85% but were often limited by cross‐sectional designs and reliance on curated texts like novels and speeches. Social media data offer a more ecologically valid, continuous assessment of linguistic changes.

This study explores whether cognitive decline can be detected through online activity on Reddit, contributing to developing a more complex model capable of detecting early changes indicative of cognitive deterioration from online activity.

**Method:**

We applied a Self‐Disclosure approach, commonly used in research for identifying self‐reports of various mental illnesses. We identified self‐reported AD cases on Reddit and extracted their posts. Data from 42 Reddit users (20 diagnosed with AD, 22 matched controls) were collected and analyzed to examine both online engagement patterns and linguistic shifts over time.

**Result:**

We analyzed the texts using an automatic scoring method based on DistilBERT. This method measures the amount of two types of details in a text: internal details (episodic information) and external details (repetitions and metacognitive statements).

A two‐way MANOVA showed a significant group effect on word usage (F(2, 4243) = 41.90, *p* < .001). Follow‐up analyses revealed that this effect was driven by internal words, with control using significantly more internal words than diagnosed individuals (F(1, 4244) = 52.78, *p* < .001; mean difference = ‐13.30). No significant differences were found for external words between the groups (F(1, 4244) = 0.09, *p* = .76).

An analysis of LIWC psychological categories found that patients and controls differed in their language. Most interestingly, compared to controls, patients displayed increased use of past tense, emotional, function, and social words. The controls used more analytic language reflecting logical, formal thinking, cognitive processes such as reasoning and analysis.

**Conclusion:**

Significant differences in online activity and language patterns between AD patients and controls suggest that analyzing social media activity can provide valuable insights into cognitive decline. By leveraging such linguistic and behavioral markers, future models may enable early detection of dementia in a scalable and non‐invasive manner.

